# Enhancement of feather degrading keratinase of *Streptomyces swerraensis* KN23, applying mutagenesis and statistical optimization to improve keratinase activity

**DOI:** 10.1186/s12866-023-02867-0

**Published:** 2023-05-30

**Authors:** Nagwa M. Abd El-Aziz, Bigad E. Khalil, Hayam Fouad Ibrahim

**Affiliations:** 1 Department of Microbial Genetics, National Research Centre, Dokki, 12622 Giza Egypt; 2 Genetics and Cytology Department, Biotechnology Research Institute, National Research Centre, 33 El Buhouth St, Cairo, 12622 Dokki Egypt

**Keywords:** Keratinase, Keratinase specific activity, 16SrDNA gene, UV, SA, H_2_O_2_, Mutant and ISSR

## Abstract

In this study, 25 actinomyces isolates were obtained from 10 different poultry farms and tested for their keratinase activity. The isolate with the highest keratinase activity was identified through molecular identification by PCR and sequencing of the 16S rRNA gene to be Streptomyces spp. and was named Streptomyces werraensis KN23 with an accession number of OK086273 in the NCBI database. Sequential mutagenesis was then applied to this strain using UV, H2O2, and SA, resulting in several mutants. The best keratinolytic efficiency mutant was designated as SA-27 and exhibited a keratinase activity of 106.92 U/ml. To optimize the keratinase expression of mutant SA-27, the Response Surface Methodology was applied using different parameters such as incubation time, pH, carbon, and nitrogen sources. The optimized culture conditions resulted in a maximum keratinase specific activity of 129.60 U/ml. The genetic diversity of Streptomyces werraensis KN23 wild type compared with five mutants was studied using Inter-simple sequence repeat (ISSR). The highest total and polymorphic unique bands were revealed in the S. werraensis KN23 and SA-18 mutant, with 51 and 41 bands, respectively. The dendrogram based on combined molecular data grouped the Streptomyces werraensis and mutants into two clusters. Cluster I included SA-31 only, while cluster II contained two sub-clusters. Sub-cluster one included SA-27, and sub-cluster two included SA-26. The sub-cluster two divided into two sub-sub clusters. Sub-sub cluster one included SA-18, while sub-sub cluster two included one group (SA-14 and S. werraensis KN23).

**Supplementary Information:**

The online version contains supplementary material available at 10.1186/s12866-023-02867-0.

## Introduction

One of the most pressing environmental pollution problems is the accumulation of keratinous wastes in slaughterhouses. Feathers, which are produced from poultry processing industries, are a significant by-product, as they make up 5–7% of chicken body weight. It is estimated that worldwide consumption of chicken generates several million tons of feathers annually, with approximately 2 million tons being generated as a by-product of the poultry industry globally [1, 2].

Despite the many applications of feathers, such as decorative applications, medical devices, fertilizer, dusters, bedding materials, and feedstock, a significant amount of feathers are still released into the environment without proper treatment. Traditional techniques of processing feathers, such as chemical processing and stem pressure cooking, may turn feathers into animal feeds, but they consume a large amount of energy and destroy some amino acids. Additionally, feathers keratin has become a source of pollutants due to its recalcitrant nature [3]. To address this issue, many researchers are interested in finding economic methods to convert feathers into value-added products [4]. Numerous studies have shown that different microorganisms can efficiently degrade feathers [5, 6].

Keratinase is an enzyme that can hydrolyze keratin. Diverse groups of microorganisms are known to produce keratinase [7], including bacteria such as Bacillus subtilis [8] and Chryseo bacterium [9], as well as actinomycetes from the Streptomyces genus [10]. Additionally, fungi such as Microsporum, Aspergillus [11], Chrysosporium [12], and Trichoderma [7] are also known to possess active keratinolytic properties. Enzymatic degradation of keratin using the enzymes isolated from bacterial cells or the bacterial culture has been proposed as a clean and inexpensive method of converting feather keratin into single amino acids and polypeptides.

Given the effectiveness of traditional mutagenesis for creating mutants that produce improved yields of several microbial enzymes, such as α-galactosidase and lipase [13], it is reasonable to assume that a similar strategy could be successfully applied to enhance the ability of keratinase-producing strains.

Response Surface Methodology (RSM) is an experimental approach that efficiently determines the optimal conditions for a multivariable approach [14, 15]. RSM is a collection of mathematical and analytical techniques that are used to design experiments, construct models, measure responses, analyze results, and explore relationships between controlled factors under experimental conditions. This methodology is particularly useful in bioprocess optimization for identifying the most favorable conditions.

Inter Simple Sequence Repeat (ISSR) markers are a PCR-based method that do not require any prior genomic information [16]. ISSR markers are highly polymorphic and can be used in studies on genetic diversity, phylogeny, gene tagging, genome mapping, and evolutionary biology [17]. Molecular markers like ISSR provide additional tools for characterizing, differentiating, and assessing genetic variation in collections. The ISSR-PCR technique of DNA fingerprinting has been employed in this work. It utilizes arbitrary, multi-loci markers generated by PCR amplification with microsatellite primers, which are quick, cost-effective, and easy to analyze [18]. Due to their high level of polymorphism and reproducibility, this fingerprinting technique is widely used, particularly in plants [17, 19], yeasts [20], and bacteria [21].

Therefore, the objective of this study was to isolate a new keratinase-producing actinomycetes strain and apply sequential mutagenesis to enhance keratinase productivity.

## Materials and methods

### Collecting samples

A total of 10 poultry waste samples were collected from 10 different farmers in Cairo, Egypt, from a depth of 5–10 cm, in sterile containers. Authors have permission for collection of samples obtained from the farm owners.

### Culture media and isolation of keratinase-producing bacteria

The basic medium used for isolation and fermentation of the feather-degrading bacteria was according to [22, 23]. Plate count (P.C) agar medium (Himedia, West Chester, Pennsylvania, USA) was used for actinomycetes growth.

### Preparation of keratin solution

Soluble keratin was prepared from chicken feathers obtained from local poultry waste according to the method of [24, 25].

### Keratinase production and enzyme assay

A pure single colony of freshly selected actinomycetes isolate, which was grown on P.C agar medium, was aseptically transferred for keratinase production using fermentation medium according to [23]. After incubation, the supernatant containing the enzyme excreted was used in quantitative keratinase assay. Keratinase enzyme activity was measured using keratin solution as a substrate. In brief, 1.0 ml of cell-free supernatant (crude enzyme) was incubated with 1 ml keratin in 0.05 M Tris–HCl buffer (pH 8.0) and incubated at 50 °C for 10 min as reported by [26]. One unit (U/ml) of keratinolytic activity was defined as an increase of corrected absorbance of 280 nm (A280) with the control for 0.01 per minute under the conditions described above and calculated by the following equation: U = 4 × n × A280/(0.01 × 10), where n is the dilution rate; 4 is the final reaction volume (ml); 10 is the incubation time (min).

### Determination of protein content and residual hydrolysates

Protein content was determined as described by [27] using bovine serum albumin as a standard. The residual hydrolysates were composed of undigested feathers and cells. The residual hydrolysate’s weight was determined according to [23].

### Molecular identification of keratinolytic actinomyces

DNA extraction: The extraction method was applied according to the manufacturer of (Applied Biotechnology Co., Egypt), the actinomyces isolate was identified based on partial sequencing of the 16S rRNA gene, using the universal primers as follow, forward primer 63f (5’- CAGGCCTAACACATGCAAGTC-3’) and reverse primer 1387R (5’- GGGCGGWGTGTACAAGGC-3’). PCR product was purified and sequenced as described previously [28]. PCR program was 95 °C for 3 min, 30 cycles of denaturation at 95 °C for 45 s, annealing at 56 °C for 45 s, and extension at 72 °C for 1 min /1kbp, final extension 72 °C for 5 min. PCR product was purified using MEGA Quick-Spin total fragment DNA purification kit as instructed by the manufacturer. Sequencing was performed by the Sanger method [29].The phylogenetic tree was constructed using MAFFT alignment [30]. https://mafft.cbrc.jp/alignment/server/phylogeny.html.

### Mutagenesis

The wild-type *S. werraensis* KN23was cultured on P.C broth medium at 37 °C for 2-5 days. Then, 10 ml of culture were centrifuged at 9000 g at 4 °C for 10 min to separate the cell biomass. Then, the cell biomass pellet was resuspended in 10 ml of sterilized saline (0.9%).

For UV-induced mutagenesis [31–33], sterile petri dishes containing 4 ml culture was exposed to UV light (UV dispensing cabinet fitted with 15-W lamps with about 90% of its radiation at 265 nm) [34]. The plates were placed 30 cm away from the center of UV light source and exposed to UV light for 60 min. Then, the treated plates were incubated in dark overnight to avoid photoreactivation.

For H_2_O_2_ and SA induced mutagenesis, 0.1% of sodium azide [35, 36] and 5 µl of 30% (v/v) hydrogen peroxide (H_2_O_2_) aqueous solution [35], were added to separate plates and incubated at 37 °C for 60 min. Then; After all mutagenesis treatments, cells were collected by centrifugation at 2800 g for 15 min, and washed with sterile saline Successive serial dilutions were prepared, and 0.1 ml aliquot suspension was spread on PC agar plates and incubated at 37 °C for 2-5 days. Then, *S. werraensis* KN23 colonies appeared were assayed for keratinase-specific activity to select high-efficiency keratinase producing mutants as reported by [23, 37].

### Optimization of keratinase activity

In two steps, statistical design studies were used to optimize the mutant *Streptomyces werraensis* S.A-27 for the production of keratinase. Statistical software Design-Expert® 6.0.8 (Stat-Ease, Minneapolis, MN, USA) was used for the experimental design and statistical analysis [38, 39]. To determine the best carbon and nitrogen sources for keratinase production, 0.5% carbon sources (glucose, sucrose, fructose, lactose, and xylose) and nitrogen sources (peptone, tryptone, malt extract, beef extract, and yeast extract) were screened in the first stage. According to Table 1 by placket Burman design, each independent component for the second stage RSM, including pH (5, 6, 7, 8, and 9), incubation duration (1, 2, and 3 days), carbon sources (sucrose), and nitrogen sources (yeast extract), was assessed at two distinct values, including the minimum and maximum levels (+ 1, 1) RSM is essentially a two factor experimental design with three levels of study for each factor. Based on 30 experimental designs, the reaction of keratinase activity was assessed. Keratinase activity was examined as the response using the general model equation of Y = β_o_ + β_1_X_1_ + β_2_X_2_ + β_3_X_3_ + β_11_X_12_ + β _22_X_22_ + β_33_X_32_ + β_12_X_1_X_2_ + β_13_X_1_X_3_ + β_23_X_2_X_3_. The significance of each coefficient in the equation was determined by Fisher’s F test and analysis of variances (p < 0.05). The quadratic models were showed as contour plots (3D). The statistical analysis of the data obtained from RSM for keratinase production was subjected to analysis of variance (ANOVA). All experiments were conducted in triplicate.

**Table 1:** Experimental factors and level of minimum and maximum range for statistical screening using Plackett–Burman factorial design (PBFD)

Factors	Independent Factor	Unit	Range Level	
			**Minimum (− 1)**	**Maximum (+ 1)**
X1	Incubation time	Hours	24	72
X2	pH	-	5	9
X3	Sucrose (carbon source)	% (w/v)	0.5	2.5
X4	Yeast extract (nitrogen source)	% (w/v)	0.5	2.5

### Molecular markers ISSR for actinomycetes wild type and mutants strains

Inter simple sequence repeats (ISSR) analysis was applied [40], and procured from UBC (University of British Columbia, biotechnology laboratory, Vancouver, Canada) based on core repeats anchored at the 5' or 3' end as in in Table 2. Six actinomycetes genotypes were tested. DNA was amplified using Taq-DNA polymerase chain reaction (PCR) as in the manufacturer’s instructions master mix (amaRonePCR) for ISSR primers, the PCR reaction consisted of a 5 min incubation period at 94 °C followed by 40 cycles of 94 °C/50 s,45 °C/1 min and 72 °C/1 min, with a final extension step of 72 °C/10 min. The PCR product was separated by 1.5% agarose gel electrophoresis using a TAE buffer and 0.004% red safe dye. The gel was photographed by gel documentation (Bio-Rad) and analyzed by Total Lab program to find out the molecular weight of each band and that to compare the presence and absence of the band among cultivars [21], and this data was imported in multi-variant statistical package (MVSP) Version 3.1 to find the similarity matrix and dendrogram.

**Table 2:** The ISSR primers name and sequences

No	Primer	Sequence
1	ISSR-152	5'- (AG)8YC-3'
2	ISSR-153	5'- (AG)8YG-3'
3	ISSR-154	5'- (AC)8YT-3'
5	UBC 851	5'- (GT)8YG-3'
6	ISSR-157	5'- CGCGATAGATAGATAGATA-3'
7	ISSR-158	5'- GACGATAGATAGATAGATA-3'
11	ISSR-861	5'- (GT)6YG-3'

(University Of British Columbia-UBC): Y = pyrimidine (C or T)

## Results

### Isolation and quantitative keratinase assay of actinomycete isolates

**Fig. 1:**
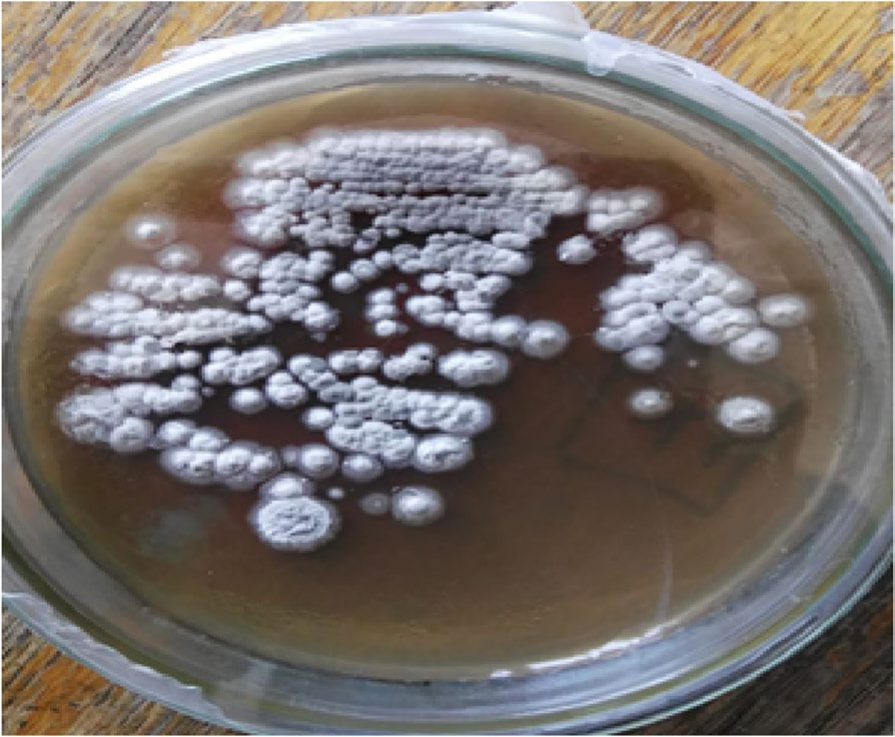
Colony morphology of Streptomyces werraensis KN23 isolate on plate count agar

A total of 25 actinomycete colonies were isolated from 10 different poultry waste samples collected from 10 different farmers in Cairo, Egypt. The serial dilution method was used to spread each sample on isolation agar medium and incubated at 37 °C for 2–5 days. Selection of actinomycetes was performed based on their morphological differences, and then stored on plate-count agar slants for further study. The obtained actinomycete isolates were assayed for keratinase production and specific activity. Results showed a broad range of keratinase specific activity. The isolate with the highest keratinase activity was isolate no. 23, which recorded 51.60 U/ml. Another keratinase activity assay was done for all isolates by determining the protein content of their residual feather, which was measured at A280, as indicated by. The isolate with the highest keratinase activity was no. 23 recorded in the basic medium, with a residual feather weight of 190 ± 0.045 mg and a protein content of 1.052 mg/ml. It was capable of completely degrading feathers within 48 h (out of 1% feather). This isolate was named KN23, as shown in Fig. 1.

### The molecular identification of actinomyces KN23 using 16S rDNA gene alignment in Genbank (Blast)

**Fig. 2:**
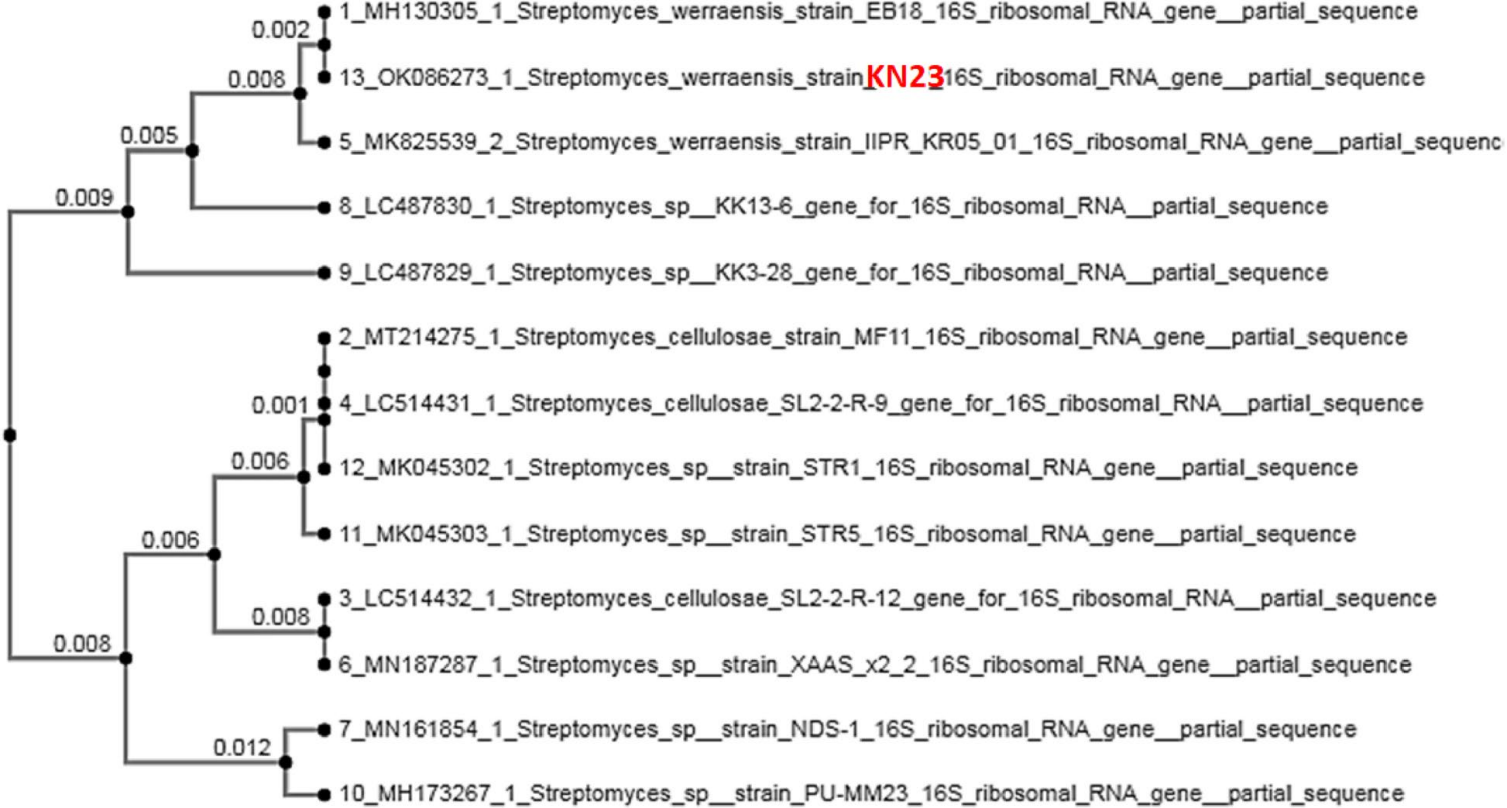
The phylogenetic tree of *Streptomyces werraensis* KN23 accession no. OK086273 and the other isolates in genbank

The nucleotide sequence of the KN23 16S rDNA gene was submitted to GenBank and produced a fragment of approximately 1171 bp, with accession number OK086273. The obtained sequence was subjected to BLAST queries using the 'blastn' algorithm implemented at NCBI to determine the putative identity of the strain (www.ncbi.nlm.nih.gov/BLAST). Based on sequence homology, the top hit on BLAST showed 100% similarity with Streptomyces werraensis (Fig. 2), leading to the conclusion that the isolate was Streptomyces werraensis KN23."

### Multistep mutagenesis of streptomyces werraensis KN23 for improved keratinase production

In the first step mutation, Streptomyces werraensis KN23 was treated with UV mutagenesis to improve its keratinase productivity. Exposure to 60 min of UV resulted in the isolation of a total of forty-five Streptomyces werraensis KN23 surviving colonies. These were assayed for keratinase specific activity, and only three mutants exhibited high efficiency in keratinase specific activity. The mutant UV-36 was the most hyperactive, recording 72.34 U/ml (about 40% more than wild type W.T), with a residual feather weight of 160 ± 0.011 mg (about 16% less than the wild type) and a protein content of 1.247 mg/ml (about 19% more than the wild type), as shown in Table 3.

**Table 3:** Estimation of keratinase specific activity produced by strain *Streptomyces werraensis KN23* and its mutants after 48 h incubation at 37 °C

*Streptomyces werraensis KN23* strain	Keratinase specific activity (U/ml)	Residual feather (mg)	Protein content (mg/ml)
**First step mutation with ultra violet (UV) mutagenesis**			
**Parent** ***S. werraensis*** **KN23 strain**	51.60	190 ± 0.045	1.052
**UV-Mutants**			
U.V-14	59.87	185 ± 0.120	1.143
U.V-36	72.34	160 ± 0.011	1.247
U.V-39	66.90	170 ± 0.320	1.182
**Second step mutation with hydrogen peroxide (H**_**2**_**O**_**2**_**)**^a^ **mutagenesis**			
**Parent UV-36**	72.34	160 ± 0.011	1.247
**H**_**2**_**O**_**2**_**Mutants**			
H-7	91.07	135 ± 0.021	1.290
H-23	76.21	155 ± 0.178	1.249
H-42	74.43	150 ± 0.190	1.253
H-46	86.90	140 ± 0.167	1.265
**Third step mutation with sodium azide (SA)**^**b**^ **mutagenesis**			
**Parent H-7**	91.07	135 ± 0.021	1.290
**S.A-Mutants**			
S.A-14	49.80	110 ± 0.032	1.220
S.A-18	38.23	120 ± 0.078	1.086
S.A-26	35.01	120 ± 0.065	0.927
S.A-26	106.92	90 ± 0.231	1.437
S.A-31	46.24	115 ± 0.007	1.160

^a^Hydrogen peroxide (H_2_O_2_) 5 µl of 30% (v/v) concentration

^b^Sodium azide (SA): 0.1% concentration

In the second step mutation, the UV-36 mutant was treated for 60 min with 5 µl of 30% (v/v) H_2_O_2_. A total of fifty-five surviving colonies were assayed for keratinase specific activity, and four mutants exhibited high efficiency in keratinase. Among them, mutant H-7 was the most hyperactive, with 91.07 U/ml (about 26% higher than its origin UV-36), a residual feather weight of 135 ± 0.021 mg (about 16% less than UV-36), and a protein content of 1.290 mg/ml (about 3% more than UV-36).

In the third step mutation, the mutant H-7 was treated with 0.1% sodium azide (SA) mutagenesis for 60 min. After treatment, thirty-six surviving colonies were assayed for keratinase specific activity, and only one mutant exhibited higher efficiency. It was isolated and named SA-27, which had 106.92 U/ml (about 17% higher than its origin H-7), a residual feather weight of 90 ± 0.231 mg (about 33% less than H-7), and a protein content of 1.437 mg/ml (about 11% more than H-7), as shown in Table 3. The mutant SA-27 had a keratinase activity of 106.92 U/ml, which was higher than the wild type S. werraensis KN23, which had a keratinase activity of 51.60 U/ml.

### Screening of significant carbon and nitrogen variables

**Fig. 3:**
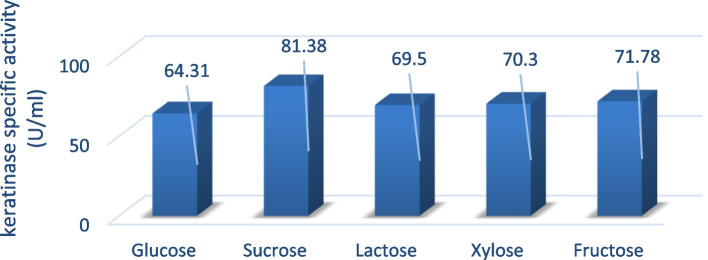
Medium optimization conditions by supplementing different carbon sources of mutant SA-27

**Fig. 4:**
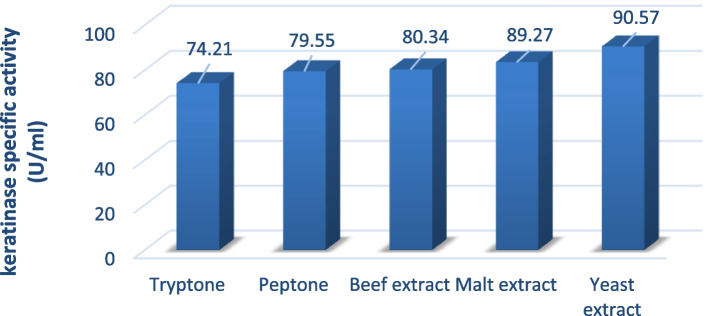
Medium optimization conditions by supplementing different nitrogen sources of mutant SA-27

The mutant SA-27 identified the significant impact of 10 carbon and nitrogen supply factors on the production of keratinase [41]. The results showed that the maximum keratinase assay was recorded in the addition of yeast extract (90.57 U/ml) and sucrose supplemented medium (81.38 U/ml), all of the variables had a positive impact on the production of keratinase [42], as demonstrated in Figs. 3 and 4.

### Optimization of keratinase activity using response surface methodology

By utilizing response surface methodology (RSM) and selecting sucrose, yeast extract, pH, and incubation time as the four variables, optimal concentrations of the medium components and incubation duration were determined. Table 4 displays the design matrix, associated findings from RSM trials, mean predicted values, and residual value [43] to assess the effects of four independent variables. The experiment involved a 30-trial matrix with four components and three levels (-1, 0, and + 1), as well as three repetitions at the central point. The results showed that 129.60 U/ml of keratinase could be produced when 1.5% yeast extract, 1.5% sucrose, pH 7, and 72 h of incubation were present.

**Table 4:** Design of different trials of the response surface methodology for independent variables and responses by actinomyces mutant SA-27

**Run**	**Factor 1** **A: ph** **H + **	**Factor 2** **B: Inc. time h**	**Factor 3** **C: sucrose** **%**	**Factor 4** **D: yeast Extract** **%**	**Actual value** **keratinase U/ml**	**Predicted Value**	**Residual**
1	7(0)	48(0)	1.5(0)	1.5(0)	109.80	113.12	-3.32
2	7(0)	48(0)	1.5(0)	0.5(-1)	101.30	106.83	-5.53
3	7(0)	48(0)	1.5(0)	1.5(0)	107.30	113.12	-5.82
4	7(0)	24(-1)	1.5(0)	1.5(0)	107.20	114.28	-7.08
5	7(0)	48(0)	1.5(0)	2.5(+ 1)	115.20	109.69	5.51
6	7(0)	48(0)	1.5(0)	1.5(0)	111.30	113.12	-1.82
7	9(+ 1)	24(-1)	2.5(+ 1)	0.5(-1)	89.23	86.65	2.58
8	7(0)	48(0)	1.5(0)	1.5(0)	113.50	113.12	0.3761
9	7(0)	48(0)	2.5(+ 1)	1.5(0)	110.70	110.01	0.6934
10	7(0)	48(0)	1.5(0)	1.5(0)	119.80	113.12	6.68
11	5(-1)	24(-1)	0.5(-1)	2.5(+ 1)	72.58	72.58	-0.0040
12	5(-1)	24(-1)	0.5(-1)	0.5(-1)	70.66	67.83	2.83
13	7(0)	72(+ 1)	1.5(0)	1.5(0)	129.60	122.54	7.06
14	9(+ 1)	72(+ 1)	0.5(-1)	0.5(-1)	98.11	101.50	-3.39
15	5(-1)	72(+ 1)	0.5(-1)	2.5(+ 1)	79.34	82.53	-3.19
16	5(-1)	24(-1)	2.5(+ 1)	0.5(-1)	72.60	75.70	-3.10
17	5(-1)	24(-1)	2.5(+ 1)	2.5(+ 1)	75.03	72.24	2.79
18	9(+ 1)	48(0)	1.5(0)	1.5(0)	93.90	95.95	-2.05
19	7(0)	48(0)	1.5(0)	1.5(0)	117.10	113.12	3.98
20	9(+ 1)	72(+ 1)	0.5(-1)	2.5(+ 1)	114.40	110.69	3.71
21	7(0)	48(0)	0.5(-1)	1.5(0)	113.60	114.31	-0.7122
22	9(+ 1)	72(+ 1)	2.5(+ 1)	2.5(+ 1)	90.76	94.20	-3.44
23	5(-1)	72(+ 1)	0.5(-1)	0.5(-1)	77.30	77.61	-0.3079
24	9(+ 1)	72(+ 1)	2.5(+ 1)	0.5(-1)	93.85	93.24	0.6126
25	5(-1)	72(+ 1)	2.5(+ 1)	2.5(+ 1)	74.17	77.55	-3.38
26	9(+ 1)	24(-1)	2.5(+ 1)	2.5(+ 1)	88.36	87.44	0.9165
27	9(+ 1)	24(-1)	0.5(-1)	2.5(+ 1)	96.37	99.30	-2.93
28	5(-1)	48(0)	1.5(0)	1.5(0)	78.44	76.40	2.04
29	9(+ 1)	24(-1)	0.5(-1)	0.5(-1)	94.26	90.28	3.98
30	5(-1)	72(+ 1)	2.5(+ 1)	0.5(-1)	83.17	80.85	2.32

**Fig. 5:**
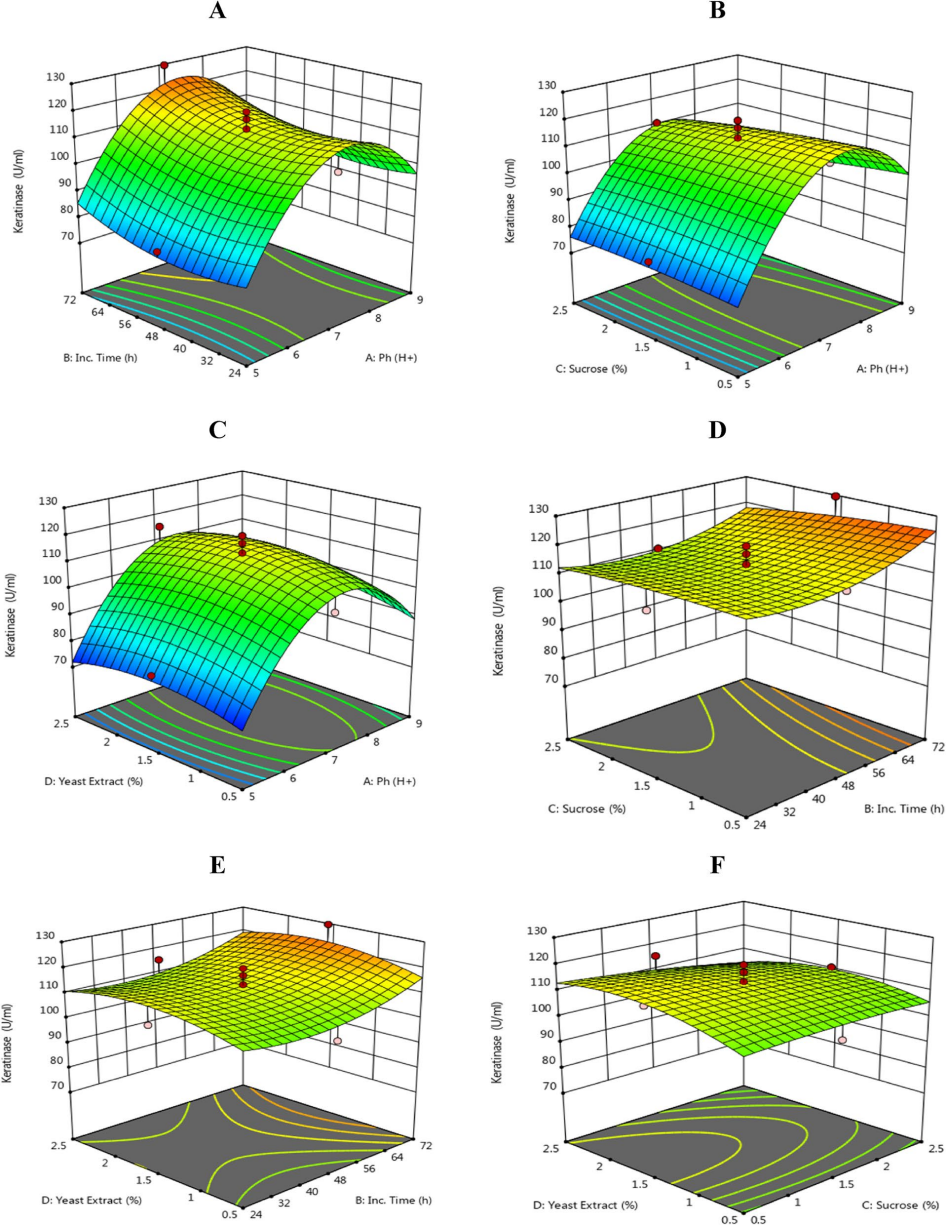
Contour plots of keratinase activity as a function of the interactions of four variables by keeping the other at centre level. **a** incubation time, pH (**b**) sucrose%, pH (**c**) yeast extract %, pH (**d**) sucrose %, incubation time (**e**), yeast extract %, incubation time, and (**f**) yeast extract % and sucrose % on keratinase production by mutant S.A-27

The quadratic regression model's analysis of variance revealed that it is highly significant with a 21.05 *F*-value (Table 5). The adjusted determination coefficient "Adj R-Squared" of 0.9063 yielded a predictive R Squared value of 0.7789, which is considered very high according to the F-test (Tables 6 and 7). The R-squared value indicates the proportion of observed response values' variability that can be explained by the experimental factors and their interactions. Response surface plots were utilized to study the effects of interactions and variable responses (Fig. 5) [44, 45].

**Table 5:** Analysis of variance (ANOVA) for Response Surface Quadratic Model CCD) by actinomyces mutant SA-27

**Source**	**Sum of Squares**	**Df**	**Mean Square**	***F*-value**	***p*-value**	
**Model**	7875.76	14	562.55	21.05	< 0.0001	significant
A-Ph	1719.91	1	1719.91	64.35	< 0.0001	
B-Inc. Time	307.60	1	307.60	11.51	0.0040	
C-Sucrose	83.42	1	83.42	3.12	0.0976	
D-Yeast Extract	36.78	1	36.78	1.38	0.2591	
AB	2.10	1	2.10	0.0784	0.7833	
AC	132.42	1	132.42	4.95	0.0418	
AD	18.17	1	18.17	0.6797	0.4226	
BC	21.51	1	21.51	0.8046	0.3839	
BD	0.0264	1	0.0264	0.0010	0.9753	
CD	67.61	1	67.61	2.53	0.1326	
A^2^	1881.02	1	1881.02	70.37	< 0.0001	
B^2^	72.38	1	72.38	2.71	0.1206	
C^2^	2.41	1	2.41	0.0902	0.7681	
D^2^	61.31	1	61.31	2.29	0.1507	
**Residual**	400.93	15	26.73			
Lack of Fit	292.12	10	29.21	1.34	0.3924	not significant
Pure Error	108.81	5	21.76			
**Cor Total**	8276.69	29

**Table 6:** Regression values by CCD

**Std. Dev**	5.17	**R**^**2**^	0.9516
**Mean**	96.63	**Adjusted R**^**2**^	0.9063
**C.V. %**	5.35	**Predicted R**^**2**^	0.7798
		**Adeq Precision**	14.9674

**Table 7:** Sequential model sum of squares

**Source**	**Sum of Squares**	**Df**	**Mean Square**	***F*-value**	***p*-value**	
Mean vs Total	2.801E + 05	1	2.801E + 05			
Linear vs Mean	2147.71	4	536.93	2.19	0.0993	
2FI vs Linear	241.83	6	40.30	0.1301	0.9909	
**Quadratic vs 2FI**	**5486.22**	**4**	**1371.55**	**51.31**	** < 0.0001**	**Suggested**
Cubic vs Quadratic	290.65	8	36.33	2.31	0.1438	Aliased
Residual	110.28	7	15.75			
Total	2.884E + 05	30	9613.44

### Adequacy of the model

To ensure the accuracy of the model, a random set of 30 production combinations was used to experimentally retest the keratinase production [44, 46]. The optimized conditions determined from the model predicted a keratinase production of 129.60 U/ml. The experimental value obtained was 122.54 U/ml, which was close to the predicted value. The model was found to be valid and reliable, as confirmed by the close agreement between the experimental and predicted values, as indicated in Table 4.

Factor coding is Coded.

Sum of squares is Type III – Partial.

The Model *F*-value of 21.05 indicates that the model is significant, and there is only a 0.01% chance that such a large *F*-value could occur due to noise.

Model terms with *P*-values less than 0.0500 are considered significant, and in this case, A, B, AC, and A^2^ are significant model terms. Model reduction may improve the model if there are many insignificant model terms (excluding those required to support hierarchy), with values greater than 0.1000.

The Lack of Fit *F*-value of 1.34 implies that the Lack of Fit is not significant relative to the pure error, and there is a 39.24% chance that a Lack of Fit *F*-value this large could occur due to noise. Non-significant Lack of Fit is desirable as it means that the model fits well.

The Predicted R^2^ of 0.7798 is in reasonable agreement with the Adjusted R^2^ of 0.9063; the difference is less than 0.2.

The Adeq Precision measures the signal to noise ratio, and a ratio greater than 4 is desirable. The ratio of 14.967 indicates an adequate signal, and this model can be used to navigate the design space.

Select the highest order polynomial where the additional terms are significant and the model is not aliased.

### Molecular depiction

**Fig. 6:**
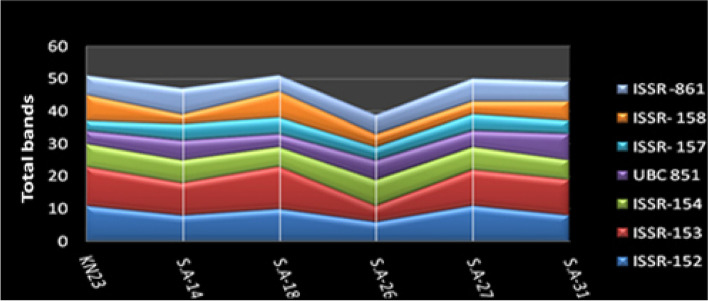
The relationship between total bands of the different primers used for the detection of the strain KN23 strain and five mutants

**Fig. 7:**
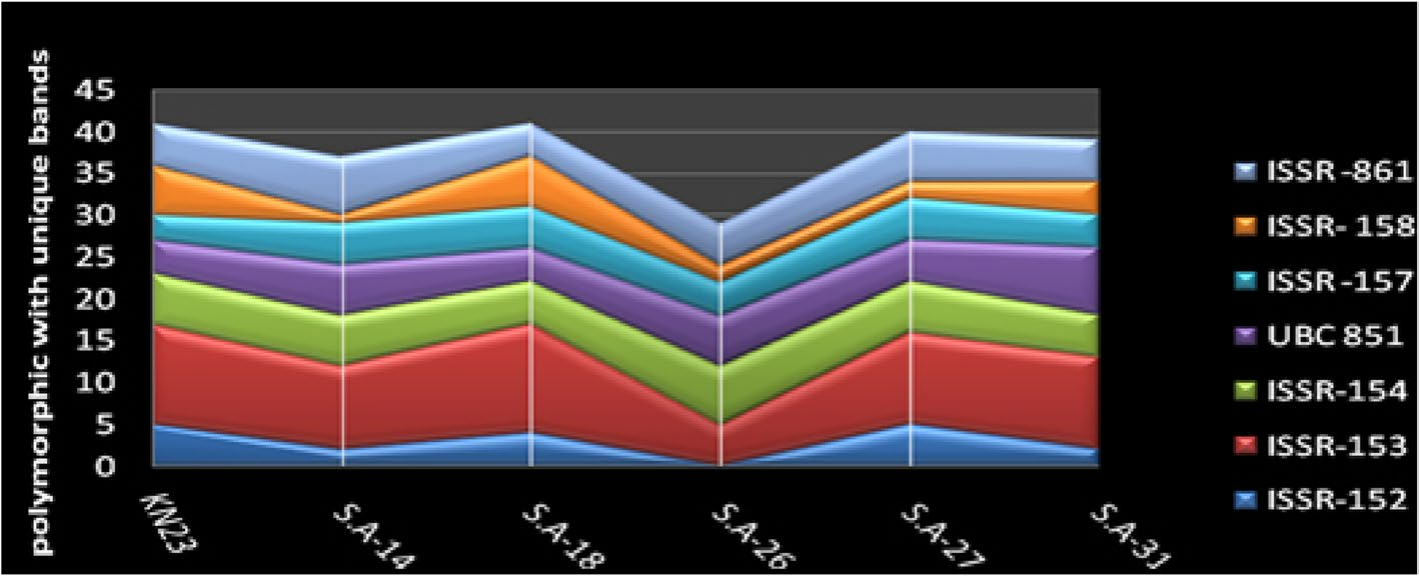
The relationship between, polymorphic with unique bands and polymorphism percentage of the seven ISSR primers used for the detection of six Streptomyces werraensis KN23 and five mutants

**Fig. 8:**
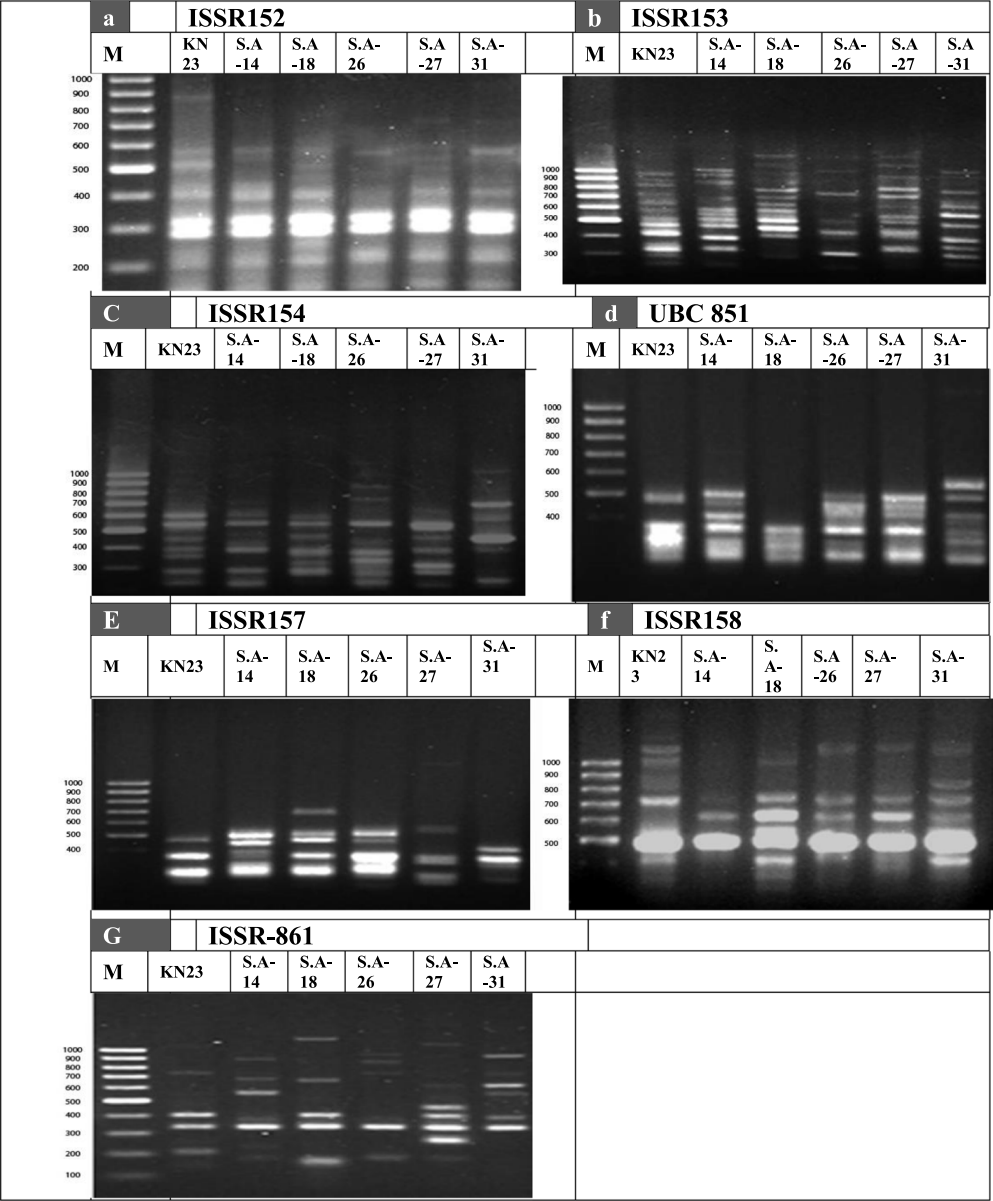
The Inter-Simple Sequence Repeats (ISSR) amplification pattern obtained for six samples of Streptomyces werraensis strain KN23 and five mutants, a primer ISSR-152, b primer ISSR-153, c primer ISSR-154, d primer UBC-851, e primer ISSR-157 f primer ISSR-158 and g primer ISSR-861

Molecular description of *S.werraensis* KN23 and its mutants were conducted using ISSR primers (ISSR-152, ISSR-153, ISSR-154, UBC851, ISSR-157, ISSR-158, and ISSR-861). The ISSR analysis produced a total of 122 markers, with 112 of them being polymorphic, and unique bands displaying 91% polymorphism, as shown in Table 8 and Fig. 7. The highest number of total bands were displayed in *S.* werraensis KN23 and S. A-18, with 51 bands, followed by the mutant S. A-27 with 50 bands, as indicated in Table 9 and Fig. 6. The number of polymorphic bands with unique markers was 41 for KN23 and SA-18, whereas the mutants S.A-27, S.A-31, and S.A-14 showed 40, 39, and 37 bands, respectively. The lowest number of total bands and polymorphic bands with unique markers were displayed in isolate SA-26, with 39 and 29 bands, respectively, compared to the other genotypes, as shown in Tables 9 and 10, and Figs. 6, 7 and 8.

**Table 8:** Band variation and polymorphism percentage in six mutants *Streptomyces werraensis* using seven ISSR primers

Primers	Total Bands	Molecular Size (bp)	Number of Monomorphic	Number of Polymorphic + uniq	Polymorphism %
ISSR-152	17	152–1268	6	11	64%
ISSR-153	28	254–1249	0	28	100%
ISSR-154	17	246–1044	1	16	94%
UBC 851	15	294–1153	0	15	100%
ISSR -157	12	292–1323	0	12	100%
ISSR- 158	12	322–1255	2	10	83%
ISSR -861	21	153–1340	1	20	95%
	122	0	10	112	91%

**Table 9:** The total amplified fragments produced by each primer from *Streptomyces werraensis* strain and its five mutants

Total bands	Primers							
Genotypes	**ISSR-152**	**ISSR-153**	**ISSR-154**	**UBC 851**	**ISSR- 157**	**ISSR- 158**	**ISSR -861**	**Total**
*S.werraensis* KN23	11	12	7	4	3	8	6	51
SA-14	8	10	7	6	5	3	8	47
SA-18	10	13	6	4	5	8	5	51
SA-26	6	5	8	6	4	4	6	39
SA-27	11	11	7	5	5	4	7	50
SA-31	8	11	6	8	4	6	6	49

**Table 10:** Polymorphic and unique amplified fragments produced by each primer from *Streptomyces werraensis*strain and its five mutants

poly + uniq.bands	ISSR-152	ISSR-153	ISSR-154	UBC 851	ISSR -157	ISSR- 158	ISSR -861	Total
*S.werraensis*KN23	5	12	6	4	3	6	5	41
SA-14	2	10	6	6	5	1	7	37
SA-18	4	13	5	4	5	6	4	41
SA-26	0	5	7	6	4	2	5	29
SA-27	5	11	6	5	5	2	6	40
SA-31	2	11	5	8	4	4	5	39

### Proximity matrix analysis (Genetic Similarity)

Data showed in Table 11 recorded (15) pairwise comparisons to debate the genetic relationships among 6 *Streptomyces werraensis* genotypes detected in terms of similarity. The genetic similarity ranged from (0.380 to 0.571) with an average of (0.475), where the biggest value of genetic similarity was (0.571) among (*S.werraensis*KN23 and SA-14) and the lowest value of similarity was (0.380) between (SA-18 and SA-31), respectively. Also, high genetic similarity values were observed within, (SA-14 and SA-18) (0.551), (*S.werraensis* KN23 and SA-18) (0.549), (SA-26 and SA-27) (0.539),While (SA-14 and SA-26) (SA-18 and SA-27) was (0.535) on the other hand (*S.werraensis* KN23 and SA-26) (0.489) (*S.werraensis*KN23 and SA-27) (0.475) (KN23 and SA-31) (0.420) finaly the (SA-14 and SA-31) (0.396), (SA-14 and SA-27) (0.392) and (SA-18 and SA-31) (0.380) respectively.

**Table 11:** Genetic similarity percentages for *Streptomyces werraensis* KN23 strain and its five mutants, using 5 ISSR Primers

	**S. werraensis KN23**	**S.A-14**	**S.A-18**	**S.A-26**	**S.A-27**	**S.A-31**
S. werraensis KN23	1.000					
SA-14	0.571	1.000				
SA-18	0.549	0.551	1.000			
SA-26	0.489	0.535	0.533	1.000		
SA-27	0.475	0.392	0.535	0.539	1.000	
SA-31	0.420	0.396	0.380	0.432	0.505	1.000

### Cluster analysis (Phylogenetic tree)

**Fig. 9:**
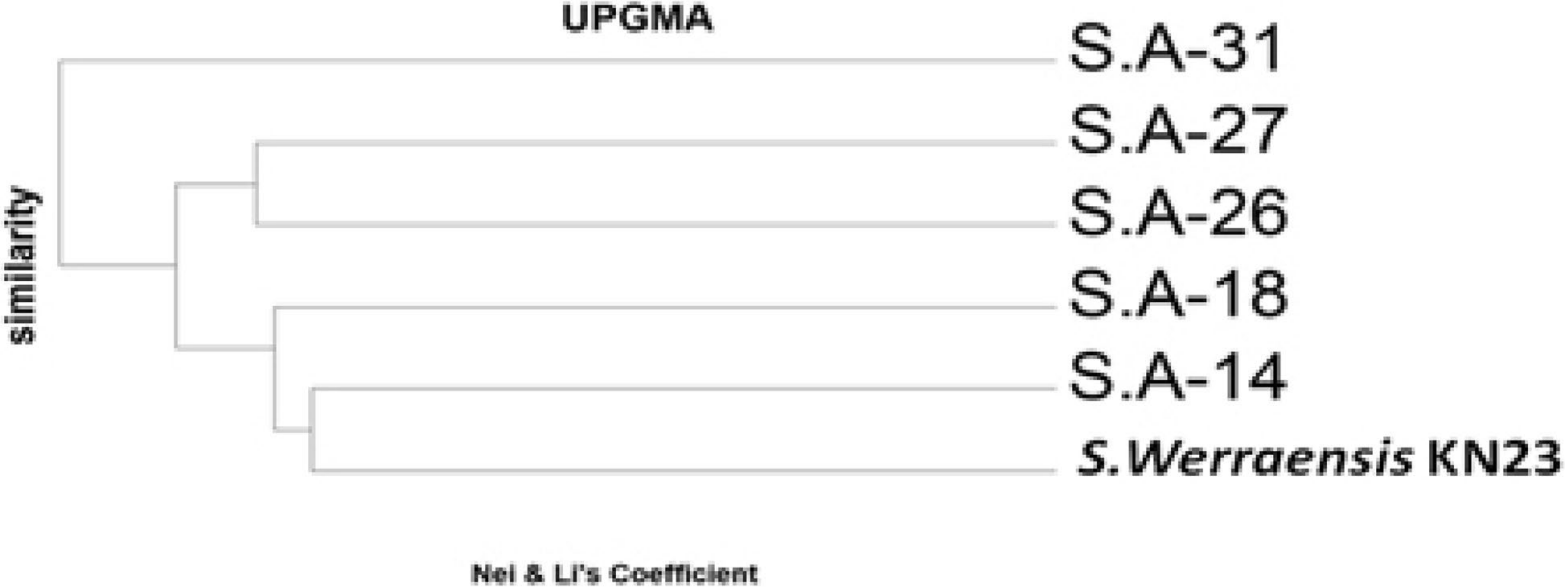
UPGMA based dendrogram showing the genetic relationship among 6 genotypes of S. werraensisusing 7 primers of ISSR

Results of cluster analysis viewed in Fig. 9 divided all *Streptomyces werraensis* genotypes into two main clusters. The cluster I included (SA-31) only while, cluster II contained two sub-clusters. The sub-cluster one included (SA-27), the sub-cluster two included (SA-26). While, the sub-cluster two divided into two sub-sub clusters. The sub-sub cluster one included (SA-18), while the sub-sub cluster two included one group (SA-14 and *S.werraensis KN23*).

## Discussion

Twenty-five Actinomyces isolates were obtained from ten different poultry farms and were tested for keratinase activity. Among these isolates, Streptomyces werraensis KN23, which was isolated from Cairo, Egypt, produced the highest amount of extracellular keratinase (51.60 U/ml) after 48 h. The strain of Streptomyces werraensis KN23 that produced the highest levels of keratinase was chosen. Mutation has been recognized as a tool for altering the DNA sequence of a specific gene, and mutagenesis is the source of genetic variants [35]. Numerous studies have been conducted to improve keratinase production using gene modification and mutation techniques. When using a conventional genetic approach to mutagenesis, random mutations are used to increase a wild strain's production of the desired metabolites [36]. Although the manufacture of industrial enzymes was significantly aided by the DNA recombinant approach, random mutagenesis is still preferred and frequently chosen as the best option due to consistent strain development [31]. The advantages of random mutagenesis using chemical mutagens are overwhelming due to their simplicity and low-cost procedure compared to recombinant DNA [35]. The improvement of bacterial enzyme production by random mutation has received considerable attention. Several methods have been included in the usage of physical mutagenesis, including Ɣ-rays, X-rays, ultraviolet (UV) rays, or chemical mutagenesis, such as Colchicine, hydrogen peroxide (H_2_O_2_), ethyl methane sulfonate (EMS), sodium azide (SA), and ethidium bromide (Eth.Br.). These have been mentioned as effective mutagens for increasing the keratinase level from producing strains [32, 33]. In this work, we reported the usage of three efficient mutagenesis methods, namely hydrogen peroxide (H_2_O_2_), sodium azide (SA), and ultraviolet (UV), as a tool to modify the original wild-type strain Streptomyces werraensis KN23 in multistep mutation induction for keratinase production improvement. This resulted in a collection of Streptomyces werraensis KN23 mutant strains, with one mutant, SA-27 at a concentration of 0.1% sodium azide treatment, giving (106.92 U/ml), compared with the wild type of Streptomyces werraensis KN23 (51.60 U/ml). Thus, the present study indicated that using H_2_O_2_, SA, and UV mutagenesis to induce mutation was in favor of keratinase production improvement. This simple method provided strains that produced more enzyme than the wild type from which it was derived.

These findings corroborate those of [47], who discovered a keratinase-producing yeast isolate known as Pichia kudriavzevii, which produced 164.04 U/ml. Highly keratinase-productive mutants were produced using a multistep mutation induction procedure with ultraviolet, ethidium bromide, and ethyl methane sulfonate (EMS) mutagenesis. The results demonstrated that EMS-37, which exhibited activity of 211.90 U/ml, was the most efficient keratinolytic mutant [32]. The maximum keratinase activity was found at pH 8–9 and 40–45 °C, utilizing white chicken feathers as the keratin substrate for 72 h, with a feather concentration of 0.5–1%. Five keratinolytic bacteria were identified from poultry farm waste. When keratinase activity was boosted using the chemical mutagen ethyl methane sulfonate (EMS) and/or the physical mutagen UV radiation, five mutants with 1.51–3.73-fold higher keratinase activity than the wild type were produced [33]. The keratinolytic activity of a newly identified Bacillus safensis and a mutant variation produced by UV treatment was tested [33]. Over the course of 120 h, the mutant produced 64.4 ± 108.5 U/mL of keratinase, while the wild-type generated 35.4 ± 50.4 U/mL. Bacillus subtilis was isolated from feather meal media using sodium nitrite and UV light. A mutant strain that can produce keratinase was found to be 75.9% more potent than the wild type [31]. Bacillus subtilis that produces keratinase was also isolated and its keratinase activity was increased via chemical mutagenesis with ethyl methane sulfonate (EMS) [48]. The best mutant found had keratinase activity of 27.44 U/ml, more than three times that of the wild type.

One of the aims of this study was to identify the optimal conditions for keratinase production by evaluating the effects of various improvement parameters. By employing this technique, we were able to enhance the environmental conditions necessary for optimal enzyme production. We used response surface methodology (RSM) to simultaneously investigate the primary and interaction effects of different environmental factors on keratinase production. The results revealed that the mutant strain SA-27 exhibited maximum keratinolytic activity at pH 7, after 72 h of incubation, with 1.5% sucrose and 1.5% yeast extract, producing 129.60 U/ml of keratinase (run 13). These findings support previous studies that have also utilized RSM to optimize enzyme production under various culture conditions.

These findings are consistent with [47]. RSM was used to optimize the culture conditions for the highly productive mutant yeast Pichia EMS-37, testing keratinase activity as a result of incubation time, pH, carbon sources, and nitrogen sources. The greatest keratinase activity was observed following response surface methodology adjustment of culture conditions for mutant EMS-37 at pH 5, 72 h of incubation period, 2.5% glucose, and 2.5% beef extract (as carbon and nitrogen sources), with an activity of 240.172 U/ml (Run3). Dutta and Banerjee [49] found that RSM can increase yield and lower the cost of enzyme production by using it to optimize the production of keratinase by Bacillus cereus. Sivakumar et al. [50] also improved the invertase production from A. niger cultivated on inexpensive agricultural wastes using this technology. Penicillium bilaiae produces an acidic protease that was optimized by [51] using the RSM approach. RSM is becoming more popular due to its aptitude for efficiently aggregating multivariable ideal conditions. RSM optimization of bioprocess approaches has been hailed as a successful method of identifying the ideal production process parameters [52]. RSM was also employed by [53] to enhance the performance of the laccase enzyme. After 5 days of optimization and incubation at 32 °C, the prospective white-rot fungus Penicillium chrysogenum showed its highest activity (7.9 U/ml) compared to the conventional approach. This procedure was more accurate.

The ISSR technique was used to study the genetic diversity and relationships between Streptomyces werraensis KN23 strain and its mutants. ISSR has also been successfully employed in phylogenetic and genetic diversity studies, as it is simple, inexpensive, and produces highly reproducible profiles. The genetic diversity of the Streptomyces werraensis KN23 wild type compared with five mutants was studied using ISSR. Molecular characterization using ISSR primers resulted in the production of a total of 122 markers, with 112 of them being polymorphic, and unique bands displaying 91% polymorphism. The highest number of total bands were displayed in S. werraensis KN23 and S.A-18 with 51 bands, followed by the mutant S.A-27 with 50 bands. The number of polymorphic unique bands were 41 in KN23 and SA-18, while the mutants S.A-27, S.A-31, and S.A-14 had 40, 39, and 37, respectively. The lowest number of total bands and polymorphic unique bands were displayed in isolate SA-26 with 39 and 29 bands, respectively, compared to the other genotypes. The dendrogram based on combined molecular data grouped the Streptomyces werraensis and mutants into two clusters. Results of cluster analysis divided all Streptomyces werraensis genotypes into two main clusters. Cluster I included (SA-31) only, while cluster II contained two sub-clusters. The sub-cluster one included (SA-27), and the sub-cluster two included (SA-26). The sub-cluster two was further divided into two sub-sub clusters. The sub-sub cluster one included (SA-18), while the sub-sub cluster two included one group (SA-14 and S. werraensis KN23).

The ISSR results are consistent with previous studies. For instance, [21] used ISSR to differentiate between probiotic lactic acid bacteria isolated from different sources of milk products. In addition, [54] studied the genetic diversity between different strains of UV Mutated Bacillus cereus bacteria for Transglutaminase production compared to wild type using ISSR. These findings are similar to [55], who studied the genetic difference between *Desulfovibrio* strains isolated from atmospheric soils in a sulfidogenic microbial community using ISSR and divided the strains into two clusters.

## Conclusion

In the current study, several actinomycetes strains were assayed for keratinase production. Among them, Streptomyces werraensis KN23 showed the highest production of keratinase enzyme, recording 51.60 U/ml. Physical Ultraviolet (UV) and chemical mutagenesis (Sodium azide (SA) and hydrogen peroxide (H_2_O_2_) were employed to increase the expression of keratinase in Streptomyces werraensis KN23. A mutant strain, SA-27, exhibited significantly higher keratinolytic activity (106.92 U/ml) than its wild-type Streptomyces werraensis KN23 (51.60 U/ml). Response surface methodology was used for optimization of the microbial enzyme production by mutant SA-27, which resulted in a synergistic combination of effective parameter interactions. The optimized conditions for keratinase enzyme production were determined as pH 7, 72 h incubation time, 1.5% sucrose and 1.5% yeast extract, with a resulting activity of 129.60 U/ml. The findings of this study demonstrate a significant increase in extracellular keratinase production by mutant SA-27, which can have several industrial applications, particularly in the food industry. ISSR markers were used to study the genetic diversity and relationships between Streptomyces werraensis KN23 strain and its mutants.

## Supplementary Information

**Additional file 1.**

## Data Availability

The datasets used and analyzed during the current study are available from the authors on reasonable request.
