# Supplementary figures and images for: Enhancement of feather degrading keratinase of Streptomyces swerraensis KN23, applying mutagenesis and statistical optimization to improve keratinase activity
Source: BMC Microbiol. 2023 May 30;23:158. doi: 10.1186/s12866-023-02867-0 (PMC10228055; doi:10.1186/s12866-023-02867-0)

Original figure 8

**A**


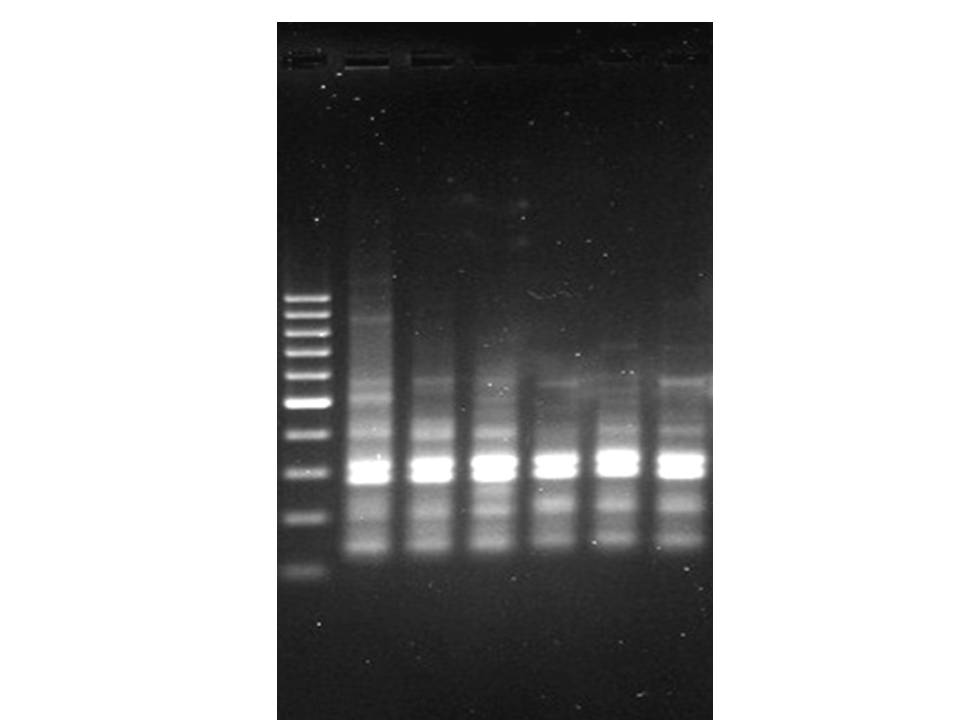


**B**


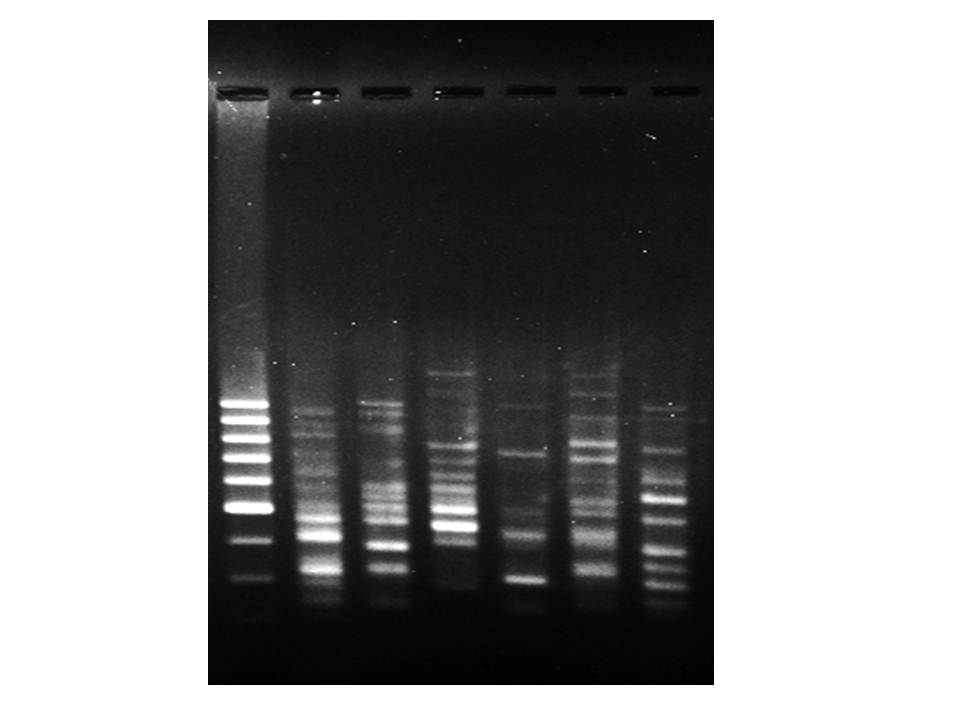


**C**


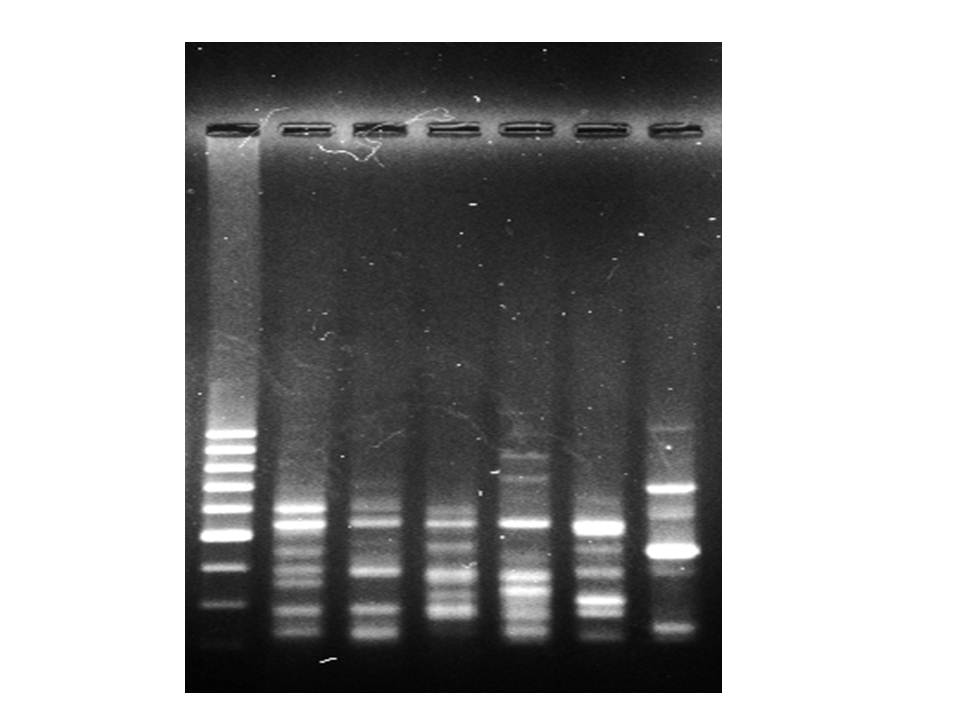


**D**


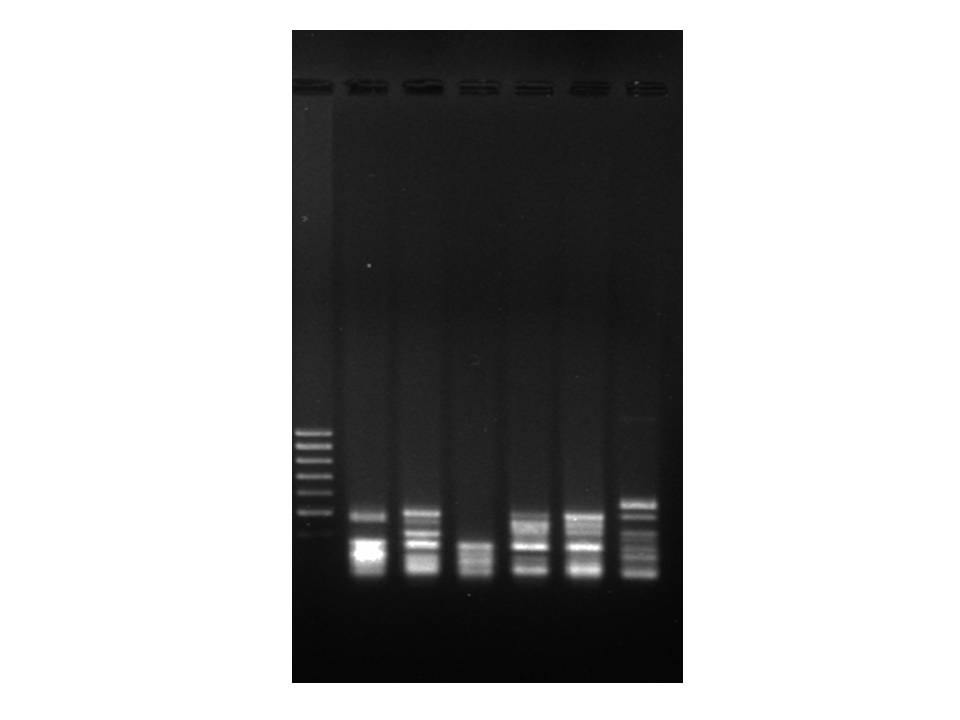


**E**


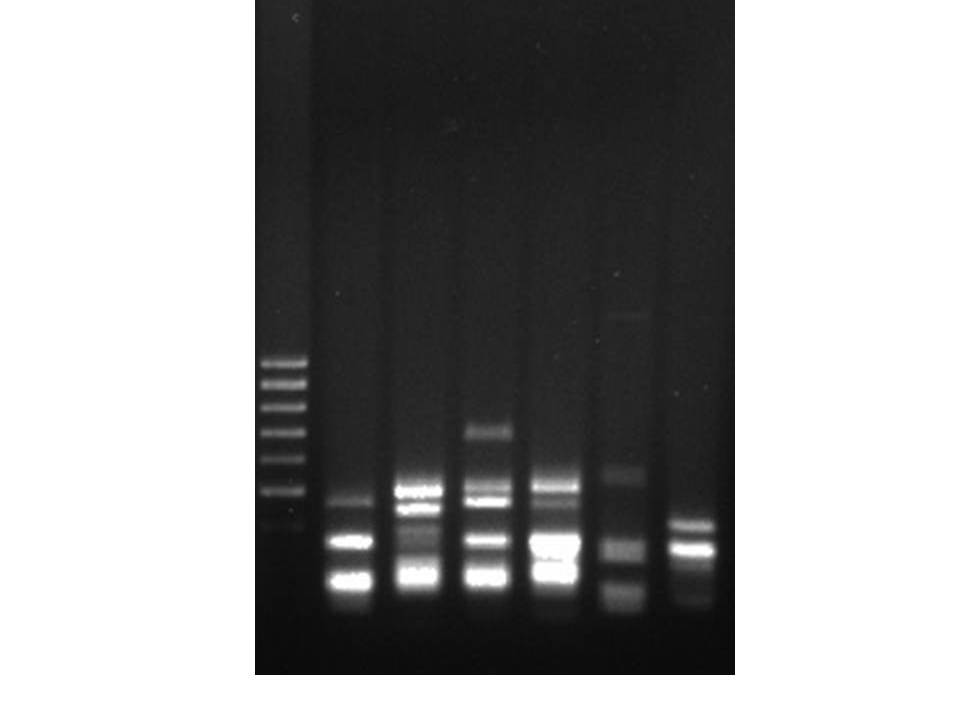


**F**


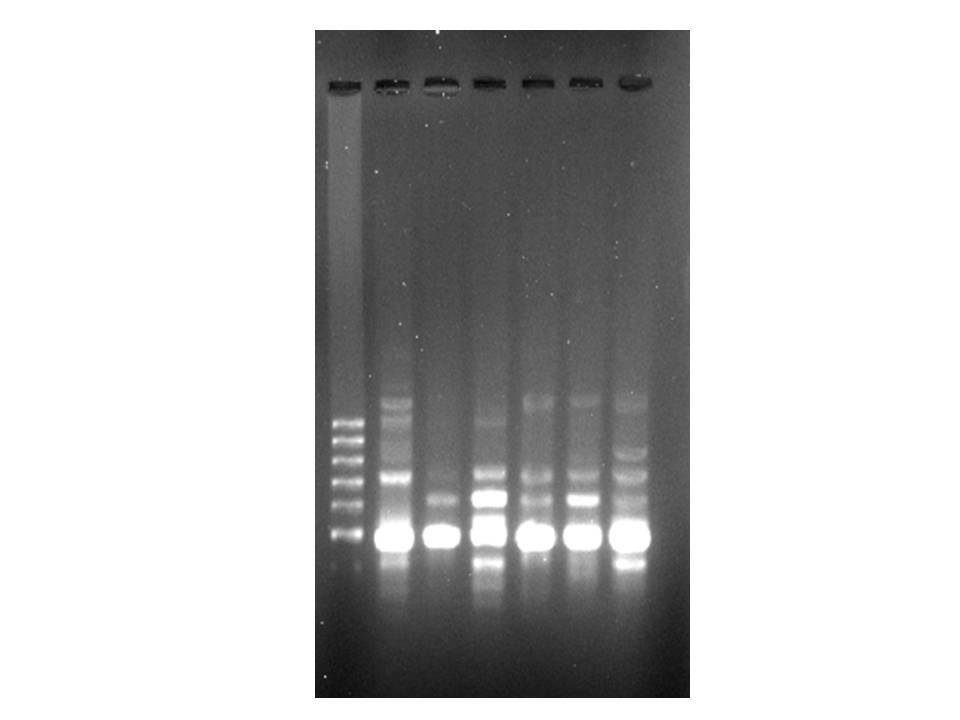


**G**


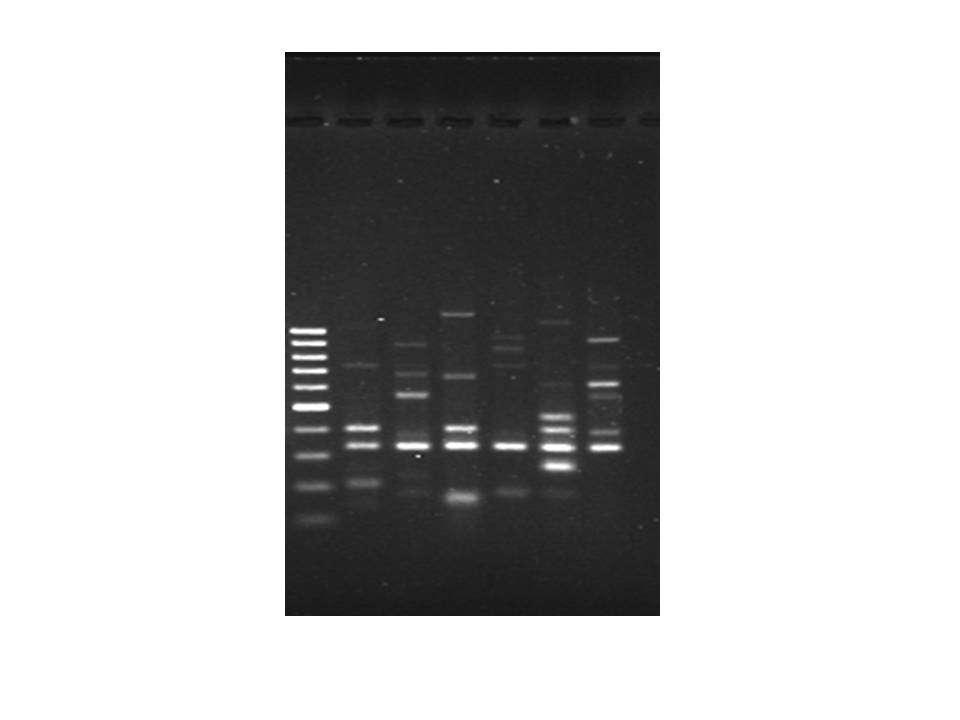

Supplement: Supplementary file 1 — Additional file 1. [file 12866_2023_2867_MOESM1_ESM.docx]
